# TRI-SCORE is superior to EuroSCORE II and STS-Score in mortality prediction following transcatheter edge-to-edge tricuspid valve repair

**DOI:** 10.1007/s00392-023-02246-9

**Published:** 2023-07-05

**Authors:** Matthias Gröger, Sophia Friedl, Dhia Ouerghemmi, Marijana Tadic, Elene Bruß, Dominik Felbel, Michael Paukovitsch, Leonhard M. Schneider, Tillman Dahme, Wolfgang Rottbauer, Sinisa Markovic, Mirjam Keßler

**Affiliations:** Department of Cardiology, Ulm University Heart Center, Albert-Einstein-Allee 23, 89081 Ulm, Germany

**Keywords:** Tricuspid regurgitation, Tricuspid valve repair, TRI-SCORE, EuroSCORE II, STS-Score

## Abstract

**Background:**

The development of transcatheter tricuspid edge-to-edge repair for tricuspid regurgitation is a therapeutic milestone but a specific periprocedural risk assessment tool is lacking. TRI-SCORE has recently been introduced as a dedicated risk score for tricuspid valve surgery.

**Aims:**

This study analyzes the predictive performance of TRI-SCORE following transcatheter edge-to-edge tricuspid valve repair.

**Methods:**

180 patients who underwent transcatheter tricuspid valve repair at Ulm University Hospital were consecutively included and stratified into three TRI-SCORE risk groups. The predictive performance of TRI-SCORE was assessed throughout a follow-up period of 30 days and up to 1 year.

**Results:**

All patients had severe tricuspid regurgitation. Median EuroSCORE II was 6.4% (IQR 3.8–10.1%), median STS-Score 8.1% (IQR 4.6–13.4%) and median TRI-SCORE 6.0 (IQR 4.0–7.0). 64 patients (35.6%) were in the low TRI-SCORE group, 91 (50.6%) in the intermediate and 25 (13.9%) in the high-risk groups. The procedural success rate was 97.8%. 30-day mortality was 0% in the low-risk group, 1.3% in the intermediate-risk and 17.4% in the high-risk groups (*p* < 0.001). During a median follow-up of 168 days mortality was 0%, 3.8% and 52.2%, respectively (*p* < 0.001). The predictive performance of TRI-SCORE was excellent (AUC for 30-day mortality: 90.3%, for one-year mortality: 93.1%) and superior to EuroSCORE II (AUC 56.6% and 64.4%, respectively) and STS-Score (AUC 61.0% and 59.0%, respectively).

**Conclusion:**

TRI-SCORE is a valuable tool for prediction of mortality after transcatheter edge-to-edge tricuspid valve repair and its performance is superior to EuroSCORE II and STS-Score.

**Graphical abstract:**

**Figure:**
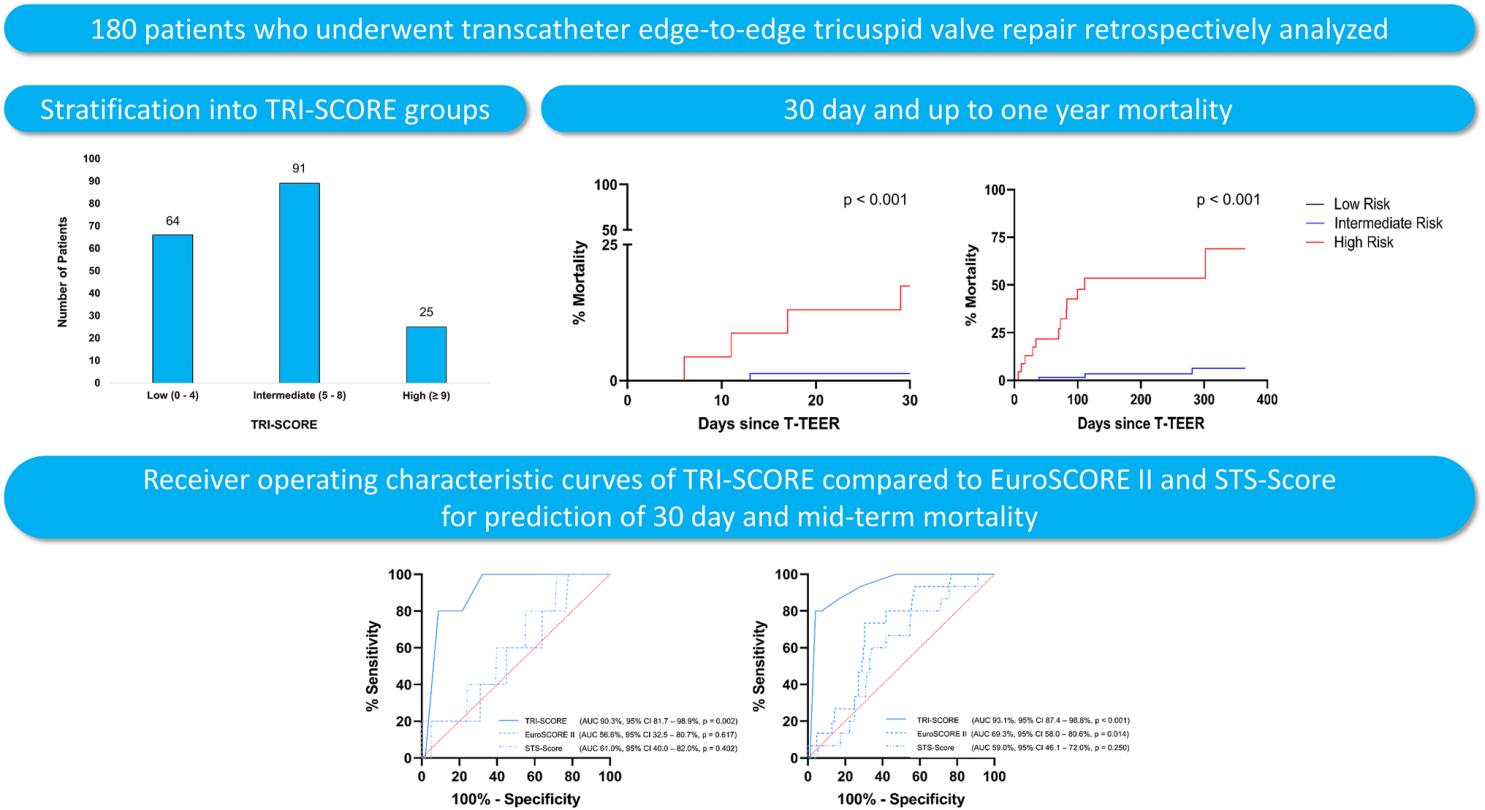
In a monocentric cohort of 180 patients undergoing edge-to-edge tricuspid valve repair TRI-SCORE predicted 30-day and up to one-year mortality more reliably than EuroSCORE II and STS-Score. *AUC* area under the curve, *95% CI* 95% confidence interval

## Introduction

Tricuspid regurgitation (TR) is a highly prevalent phenomenon in patients with heart failure (HF) [1–3]. In most cases, TR develops secondary to annular dilation or leaflet tethering [1]. The most common cause of TR is left-sided HF with subsequent postcapillary pulmonary hypertension [1]. In other cases, secondary TR can result from pure atrial dilation caused by long-standing atrial fibrillation or diastolic HF [1]. Across all HF subtypes, an estimated 20% of patients are diagnosed with significant (≥ moderate) TR [1]. Studies have shown a marked increase in mortality for HF patients suffering from TR [2, 3]. Whether TR itself or rather the underlying myocardial pathology is the driving force of adverse outcomes in these patients however is a matter of debate [2, 4–6]. Management options for TR include treatment of the underlying condition such as guideline-directed HF therapy as well as tricuspid valve repair. Since treatment of high-grade TR often implies a very high or even prohibitive surgical risk, the importance of tricuspid transcatheter edge-to-edge repair (T-TEER) continues to grow [7, 8]. Registry and case–control studies have shown a reduction in both mortality and HF-induced hospitalizations (HFH) after successful T-TEER [9–11]. A decision regarding surgical versus interventional treatment of TR should be made consensually by the heart team [12]. Clinical scoring systems such as the EuroSCORE II or the STS-Score are used for perioperative risk assessment [13, 14]. However, until recently no scoring system has been specifically validated for the complex characteristics of tricuspid valve repair. In September 2021, Dreyfus et al. introduced a risk score model for the prediction of in-hospital mortality after tricuspid valve surgery: TRI-SCORE [15]. The score consists of eight easily assessable items and includes markers of right ventricular (RV) dysfunction. TRI-SCORE has already shown good prognostic performance in patients undergoing TEER [16]. We aimed to apply the surgical TRI-SCORE model on patients undergoing T-TEER, to analyze its predictive value regarding adverse outcomes and compare its performance with the commonly used EuroSCORE II and STS-Score.

## Methods

180 patients who underwent T-TEER for severe TR at our center from March 2017 to July 2022 were consecutively included in this retrospective analysis. All patients included in the present study were symptomatic in terms of HF (New York Heart Association (NYHA) functional class ≥ II) despite guideline-directed medical therapy.

Echocardiographic characteristics at baseline were available for all study patients. Complete invasive hemodynamic parameters from right heart catheterization were available in 119 patients (66.1%). Pulmonary hypertension was classified according to the 2015 ESC/ERC guidelines [20]. TR at baseline was assessed quantitatively by transesophageal echocardiography using the five-grade system [21]. Biplane vena contracta (VC) and effective regurgitant orifice area (EROA) as estimated by the proximal isovelocity surface area method were measured [12]. Left-ventricular ejection fraction (LV-EF) was measured using the biplane Simpson's method [22].

RV function was evaluated by tricuspid annular plane systolic excursion (TAPSE). Post-procedural TR severity was assessed by 2D transesophageal echocardiography after the final device placement and removal of the guide catheter. In addition to TR severity, tricuspid valve gradients were assessed before and after clip deployment and after the removal of the guide catheter. Device success was defined as clip implantation with a reduction of tricuspid regurgitation of at least one degree at the time of discharge. TR severity at discharge was analyzed by transthoracic echocardiography.

TRI-SCORE was calculated as previously published [15]. The score consists of the following 8 risk factors with a maximum score result of 12 points: age of 70 years or higher, NYHA functional class III or IV, LV-EF of less than 60% and moderate or severe RV dysfunction (TAPSE < 17 mm and/or a Doppler tissue imaging peak systolic annular velocity *S*’ < 9.5 cm/s and/or a markedly reduced fractional area change based on a visual assessment) each contribute one point to the score. Right-sided HF signs (marked peripheral edema, ascites, severe jugular vein distension), the daily dose of furosemide of 125 mg or higher, glomerular filtration rate (GFR) of less than 30 ml/min and elevated total bilirubin each contribute two points. GFR was estimated using the Cockcroft-Gault-Formula. For elevated bilirubin, a cutoff of > 21 µmol/l was chosen according to assay-specific standards. Data collection was performed retrospectively from medical records. A complete dataset of TRI-SCORE risk factors was available for all patients. Due to the low overall event rate patients were classified into three groups according to their total score: low-risk (score 0–4), intermediate-risk (score 5–8) and high-risk (score 9–12) groups.

The primary study endpoint was all-cause mortality through a 30-day and up to one-year follow-up period. Follow-up was performed either by scheduled ambulatory visits or telephone interview.

Statistical analysis was performed using SPSS 28 software (IBM Corp., Armonk, USA). Categorical variables are expressed as counts and percentages. Normal distribution was examined using Kolmogorow–Smirnow test. Continuous parameters, if normally distributed, are presented as the mean ± standard deviation. Not normally distributed parameters are presented as the median with the interquartile range (IQR). Statistical testing of continuous variables between the three risk groups was carried out using Kruskal–Wallis test. Mortality was analyzed using Kaplan–Meier analysis and log-rank test. The sensitivity and specificity of the score were assessed via receiver operating characteristics (ROC) analysis. The degree of separability was determined through AUC (area under the curve) assessment. The diagnostic performance of TRI-SCORE, EuroSCORE II and STS-Score was compared using risk reclassification analysis. For this assessment, EuroSCORE II and STS-Score were stratified into low-risk (score < 4%), intermediate-risk (4–8%) and high-risk (> 8%) groups according to classification in the literature [12, 17, 18]. Due to the low overall event rate, the absolute net reclassification index (NRI) is shown [19].

Differences were considered statistically significant when *p* < 0.05.

The study was ethically approved and received proper oversight by the ethics committee of the University of Ulm (reference number 142/20). It complied with the principles outlined in the Declaration of Helsinki [23].

## Results

### Baseline characteristics of the study cohort and TRI-SCORE calculation

One hundred eighty patients underwent T-TEER at our center between March 2017 and July 2022. At baseline, 51 patients (28.3%) had TR grade V (torrential), 88 (48.9%) grade IV (massive) and 41 (22.8%) grade III (severe). Most patients had functional TR (86.7%). 89 patients (49.4%) were female and the median age at the time of T-TEER was 80.0 years (IQR 74.0–83.0). Patients were highly symptomatic in terms of HF: 18 patients (10.0%) had dyspnea at rest (NYHA functional class IV), 124 patients (68.9%) were in NYHA class III and 38 patients (21.1%) in NYHA class II. Patients had severe comorbidities such as renal failure [median glomerular filtration rate (GFR) 40.0 ml/min (IQR 30.0–54.0)], atrial fibrillation (87.8%) and chronic lung disease (11.1%). Median LV-EF was 50.0% (IQR 40.0–56.8) and pulmonary hypertension was frequent [median mean pulmonary artery pressure (mPAP) 31.0 mmHg (IQR 25.0–37.5)]. Most patients were classified as having isolated post-capillary pulmonary hypertension. Concomitant moderate or severe mitral regurgitation (MR) was present in 39 patients (21.7%). The high surgical risk was reflected by a median EuroSCORE II of 6.4% (IQR 3.8–10.1) and a median STS-Score of 8.1% (IQR 4.6–13.4%).

**Fig. 1:**
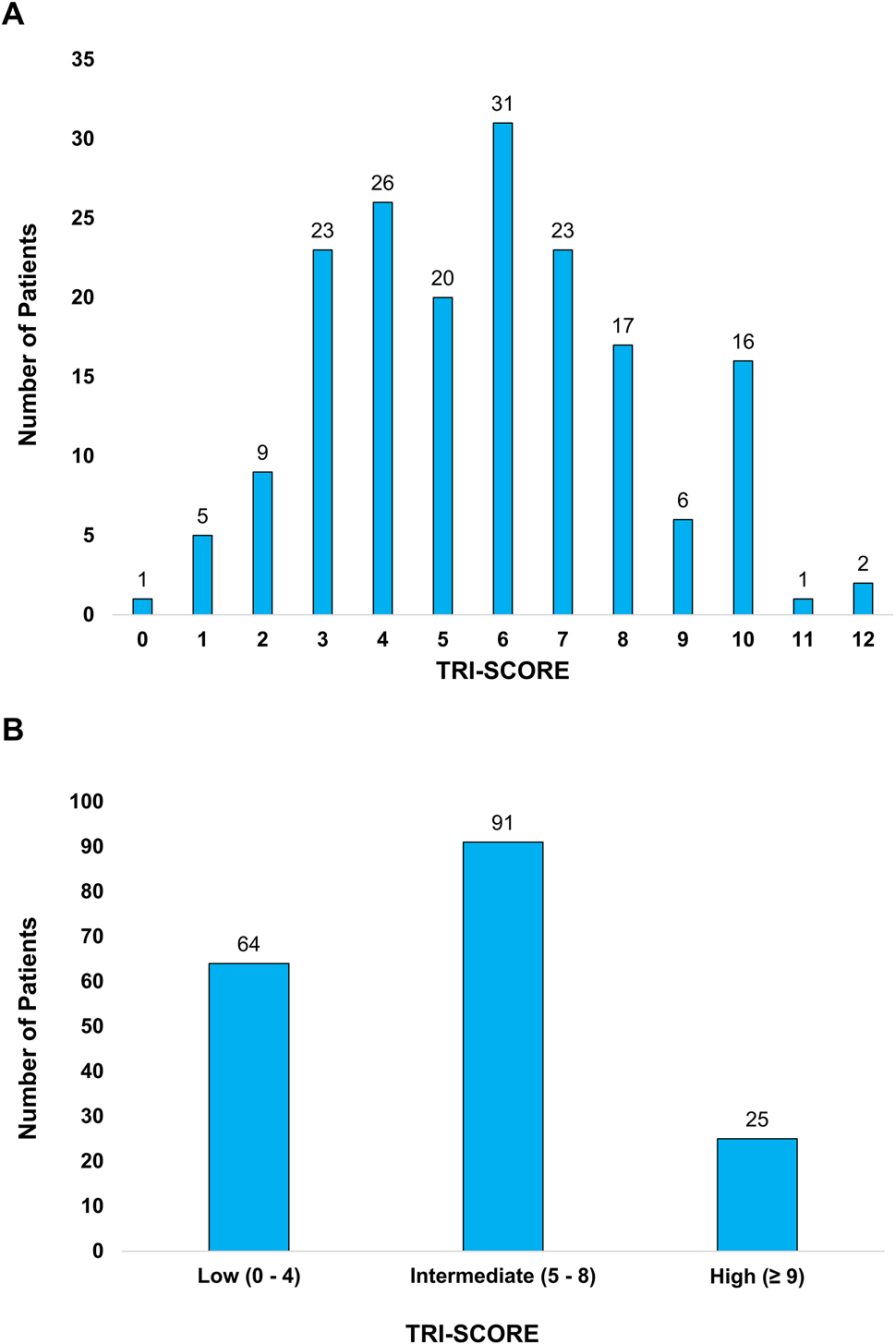
Number of patients with respective TRI-SCOREs (**A**) and within TRI-SCORE risk groups (**B**)

Median TRI-SCORE was 6.0 (IQR 4.0 – 7.0) with most patients fulfilling score criteria for age ≥ 70 years (87.2%), NYHA functional class ≥ III (78.9%) and LV-EF < 60% (78.9%). Sixty-four patients (35.6%) were classified as low-risk, 91 (50.6%) as intermediate-risk and 25 (13.9%) as high-risk groups. Group sizes with respective TRI-SCOREs are shown in Fig. 1. With the exception of age and NYHA functional class, all components of the TRI-SCORE were more pronounced in the high-risk group.

When comparing baseline characteristics between the respective TRI-SCORE risk groups significant differences were found: in the high-risk group kidney function as assessed by GFR was lower, levels of Troponin T, NT-proBNP and Bilirubin were higher, LV-EF was lower and pulmonary hypertension was more severe.

Baseline patient characteristics of the whole cohort and by TRI-SCORE risk group are shown in Table 1.

**Table 1:** Baseline characteristics of the whole cohort and by TRI-SCORE risk group

Variable	Whole cohort (*n* = 180)	Low risk (*n* = 64)	Intermediate risk (*n* = 91)	High risk (*n* = 25)	*p*
Age (years) (*n* = 180)	80.0 (74.0–83)	78.0 (73.0–81.0)	80.0 (76.0–84.0)	81.0 (74.0–84.5)	0.09
Body Mass Index (kg/m^2^) (*n* = 180)	25.9 (23.2–29.7)	26.0 (22.7–29.0)	25.3 (22.7–29.8)	27.7 (24.7–31.7)	0.21
Female sex	89/180 (49.4%)	37/64 (57.8%)	41/91 (45.1%)	9 (36.0%)	0.28
TRI-SCORE	6.0 (4.0–7.0)	3.0 (3.0–4.0)	6.0 (6.0–7.0)	10.0 (9.5–10.0)	** < 0.001**
Age ≥ 70 years	157/180 (87.2%)	55/64 (85.9%)	78/91 (85.7%)	24/25 (96.0%)	0.37
NYHA functional class ≥ III	142/180 (78.9%)	48/64 (75.0%)	71/91 (78.0%)	23/25 (92.0%)	0.20
Signs of right heart failure	92/180 (51.1%)	7/64 (10.9%)	61/91 (67.0%)	24/25 (96.0%)	** < 0.001**
Daily dose of furosemide ≥ 125 mg/torasemide ≥ 30 mg	73/180 (40.6%)	1/64 (1.6%)	48/91 (52.7%)	24/25 (96.0%)	** < 0.001**
Glomerular filtration rate < 30 ml/min	44/180 (24.4%)	2/64 (3.1%)	25/91 (27.5%)	17/25 (68.0%)	** < 0.001**
Elevated total bilirubin	34/180 (18.9%)	2/64 (3.1%)	19/91 (20.9%)	13/25 (52.0%)	** < 0.001**
Left-ventricular ejection fraction < 60%	142/180 (78.9%)	40/64 (62.5%)	78/91 (85.7%)	24/25 (96.0%)	** < 0.001**
Right ventricular dysfunction	101/180 (56.1%)	29/64 (45.3%)	50/91 (54.9%)	22/25 (88.0%)	**0.001**
NYHA functional class					0.07
Class I	0/180	0/64	0/91	0/25	
Class II	38/180 (21.1%)	16/64 (25.0%)	20/91 (22.0%)	2/25 (8.0%)	
Class III	124/180 (68.9%)	46/64 (71.9%)	60/91 (65.9%)	18/25 (72.0%)	
Class IV	18/180 (10.0%)	2/64 (3.1%)	11/91 (12.1%)	5/25 (20.0%)	
Glomerular filtration rate (ml/min) (*n* = 180)	40.0 (30–54)	50.0 (40.5–65.8)	36.0 (29.0–51.0)	27.0 (20.0–33.0)	** < 0.001**
Creatinine (µmol/l) (*n* = 180)	131.5 (98.25–166.0)	105.5 (79.3–127.8)	140.0 (111.0–174.0)	181.0 (144.0–215.5)	** < 0.001**
Bilirubin (µmol/l) (*n* = 180)	12.0 (9.0–19.0)	11.0 (8.0–14.8)	13.0 (9.0–20.0)	22.0 (9.0–28.5)	**0.01**
Troponin T (ng/l) (*n* = 179)	32.0 (21.0–55.0)	21.0 (12.0–32.0)	39.0 (24.0–56.0)	64.0 (40.0–82.5)	** < 0.001**
NT-proBNP (pg/ml) (*n* = 180)	2,665.50	1,592.50	3,693.00	3,880.00	** < 0.001**
	(1,279.0–5,055.0)	(829.8–3,226.0)	(1,636.0–6,343.0)	(1,674.0–8,281.0)	
Left-ventricular ejection fraction (%) (*n* = 180)	50.0 (40.0–56.75)	55.0 (46.3–61.8)	49.0 (40.0–55.0)	45.0 (31.0–54.0)	** < 0.001**
Baseline tricuspid regurgitation severity					0.19
Grade < I	0/180	0/64	0/91	0/25	
Grade I	0/180	0/64	0/91	0/25	
Grade II	0/180	0/64	0/91	0/25	
Grade III	41/180 (22.8%)	19/64 (29.7%)	18/91 (19.8%)	4/25 (16.0%)	
Grade IV	88/180 (48.9%)	33/64 (51.6%)	44/91 (48.4%)	11/25 (44.0%)	
Grade V	51/180 (28.3%)	12/64 (18.8%)	29/91 (31.9%)	10/25 (40.0%)	
Etiology of tricuspid regurgitation					0.24
Functional	156/180 (86.7%)	53/64 (82.8%)	81/91 (89.0%)	22/25 (88.0%)	
Degenerative	8/180 (4.4%)	5/64 (7.8%)	3/91 (3.3%)	0/25	
Pacemaker-induced	11/180 (6.1%)	5/64 (7.8%)	3/91 (3.3%)	3/25 (12.0%)	
Mixed	5/180 (2.8%)	1/64 (1.6%)	4/91 (4.4%)	0/25	
Baseline tricuspid biplane vena contracta (cm) (*n* = 179)	1.2 (1.0–1.5)	1.1 (0.9–1.4)	1.2 (1.0—1.5)	1.3 (1.0–1.7)	0.33
Baseline tricuspid EROA (cm^2^) (*n* = 83)	0.6 (0.5–0.8)	0.6 (0.5–0.8)	0.6 (0.5–0.8)	0.6 (0.6–0.8)	0.41
Baseline tricuspid septal-lateral diameter (cm) (*n* = 179)	4.4 (3.9–5.0)	4.2 (3.7–4.8)	4.6 (4.0–5.1)	4.4 (3.9–5.1)	0.057
Baseline tricuspid antero-posterior diameter (cm) (*n* = 179)	4.3 (3.8–5.1)	4.0 (3.6–4.9)	4.4 (3.8–5.1)	4.4 (3.9–5.2)	0.16
Mitral regurgitation severity					0.47
No MR	11/163 (6.7%)	6/64 (10.2%)	4/91 (4.8%)	1/25 (5.0%)	
Mild MR	113/163 (69.3%)	44/64 (74.6%)	57/91 (67.9%)	12/25 (60.0%)	
Moderate MR	30/163 (18.4%)	7/64 (11.9%)	18/91 (21.4%)	5/25 (25.0%)	
Severe MR	9/163 (5.5%)	2/66 (3.4%)	5/91 (6.0%)	2/25 (10.0%)	
Systolic pulmonary artery pressure (mmHg) (*n* = 128)	48.6 ± 12.7	42.6 ± 10.2	50.5 ± 12.5	56.7 ± 13.1	** < 0.001**
Mean pulmonary artery pressure (mmHg) (*n* = 129)	31.0 (25.0–37.5)	28.0 (23.0–33.0)	31.0 (26.0–39.0)	36.0 (30.0–42.0)	**0.001**
Pulmonary hypertension class					0.06
No PH	23/119 (19.3%)	13/43 (30.2%)	9/59 (15.3%)	1/17 (5.9%)	
Precapillary PH	1/119 (0.8%)	0/64	0/64	1 (5.9%)	
IpCPH	88/119 (73.9%)	27/43 (62.8%)	47/59 (79.7%)	14/17 (82.4%)	
CpCPH	7/119 (5.9%)	3/43 (7.0%)	3/59 (5.1%)	1/17 (5.9%)	
EuroSCORE II (%) (*n* = 180)	6.4 (3.8–10.1)	4.7 (2.7–7.2)	7.0 (4.3–10.6)	9.2 (7.0–13.6)	** < 0.001**
Extracardiac arteriopathy	23/180 (12.8%)	10/64 (15.6%)	10/91 (11.0%)	3/25 (12.0%)	0.69
Prior cardiac surgery	23/180 (12.8%)	6/64 (9.4%)	11/91 (12.1%)	6/25 (24.0%)	0.17
Diabetes mellitus on insulin	5/180 (2.8%)	1/64 (1.6%)	3/91 (3.3%)	1/25 (4.0%)	0.75
Need for hemodialysis	3/180 (1.7%)	0/64	3/91 (3.3%)	0/25	0.23
STS-Score (%) (*n* = 180)	8.1 (4.6–13.4)	6.4 (3.9–12.3)	8.9 (5.1–13.8)	10.8 (5.7–15.4)	0.063
History of cancer	21/180 (11.7%)	8/64 (12.5%)	9/91 (9.9%)	4/25 (16.0%)	0.68
Coronary artery disease	93/180 (51.7%)	28/64 (43.8%)	53/91 (58.2%)	12/25 (48.0%)	0.19
Chronic lung disease (obstructive or restrictive)	20/180 (11.1%)	5/64 (7.8%)	12/91 (13.2%)	3/25 (12.0%)	0.57
Atrial fibrillation	158/180 (87.8%)	56/64 (87.5%)	79/91 (86.8%)	23/25 (92.0%)	0.78
Procedural success	176/180 (97.8%)	63/64 (98.4%)	89/91 (97.8%)	24/25 (96.0%)	0.78
Number of implanted devices (*n* = 180)	2.0 (1.0–2.0)	1.0 (1.0–2.0)	2.0 (1.0–2.0)	2.0 (1.0–2.0)	0.12
Postinterventional mean tricuspid valve gradient (mmHg) (*n* = 174)	2.0 (1.0–2.0)	2.0 (1.0–2.0)	2.0 (1.0–2.0)	2.0 (1.0–3.0)	0.67

Bold values are statistically signficant p < 0.05

*EROA* effective regurgitant orifice area, *MR* mitral regurgitation, *NYHA* New York Heart Association, *PH* pulmonary hypertension, *IpCPH* isolated postcapillary pulmonary hypertension, *CpCPH* combined pre- and postcapillary pulmonary hypertension

### Acute procedural outcome

**Fig. 2:**
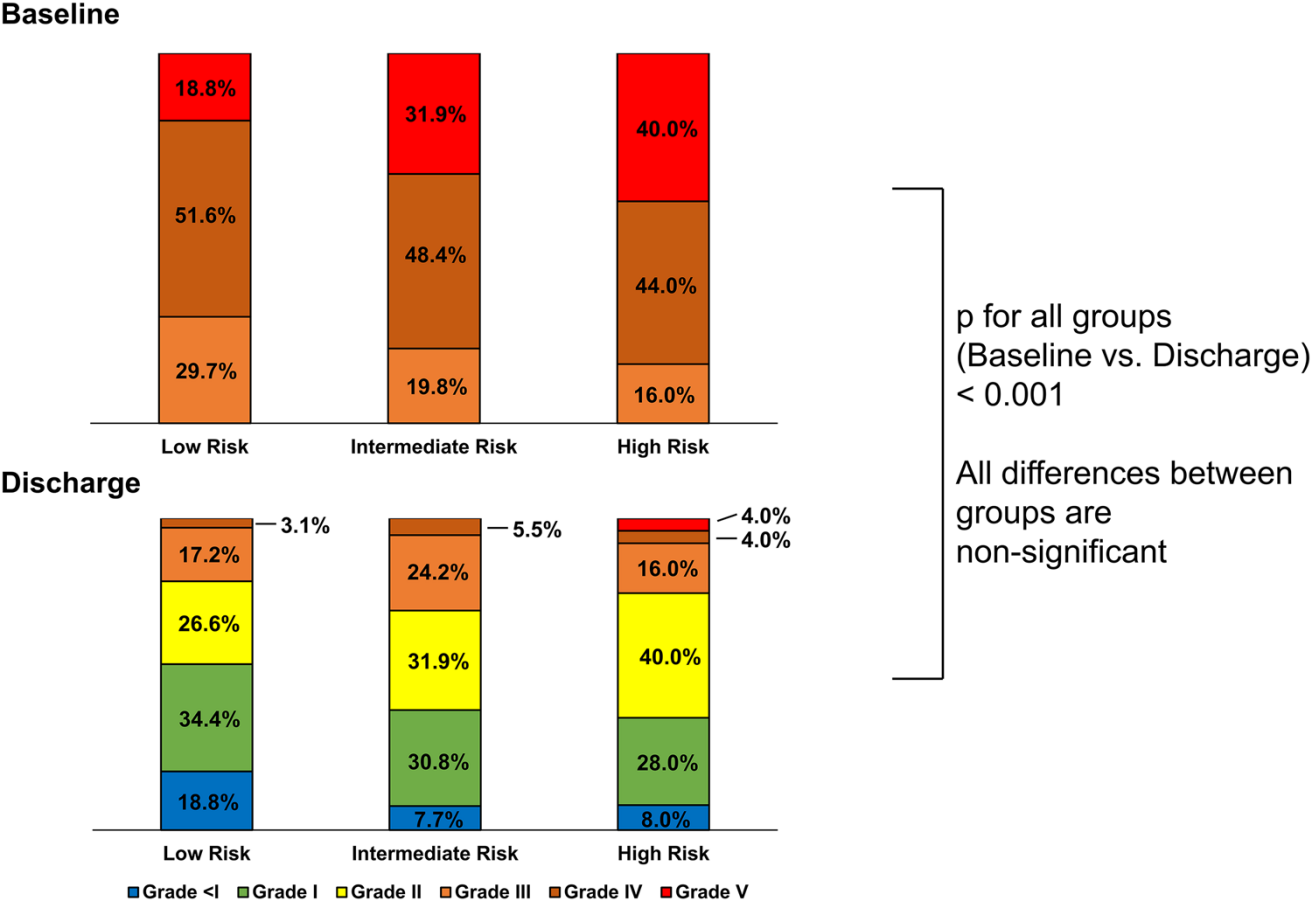
Severity of TR before T-TEER and at discharge. *TR* tricuspid regurgitation, *T-TEER* transcatheter edge-to-edge tricuspid valve repair

The procedural success rate was high at 97.8%. The median number of implanted devices was 2.0 (IQR 1.0–2.0). Devices used were off-label MitraClip (21.3%), TriClip (27.5%) and PASCAL (50.6%). Overall, 74.5% of patients had TR grade II or lower at discharge (*p* < 0.001 compared to baseline). No significant difference in TR grade at baseline or at discharge was seen between the three TRI-SCORE risk groups (Fig. 2). No intraprocedural death was recorded.

### 30-day and mid-term outcome

A 30-day follow-up was available for 163 patients. Within 30 days, no deaths occurred in the low-risk group.

**Fig. 3:**
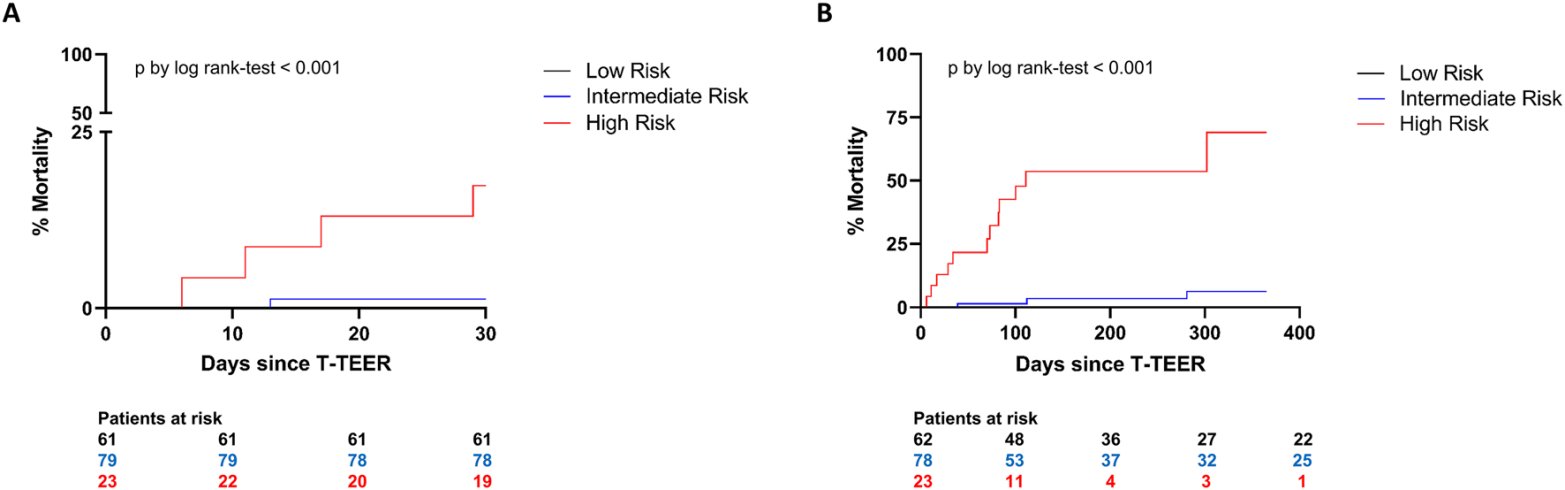
30-day mortality (**A**) and one-year mortality (**B**) in respective TRI-SCORE risk groups. *T-TEER* transcatheter edge-to-edge tricuspid valve repair

One patient in the intermediate-risk group (1.3%) and four patients in the high-risk group (17.4%) died during 30 days following the procedure (*p* < 0.001 by log-rank test). A 30-day outcome is shown in Fig. 3A.

Follow-up was recorded up to 1 year after the procedure. The median mid-term follow-up period for all study patients was 168 days. During this time, no deaths were registered in the low-risk group, three deaths in the intermediate-risk group (3.8%) and 12 deaths in the high-risk group (52.2%) (*p* < 0.001 by log-rank test). The main cause of death was cardiovascular (11 patients, 73.3%). No device-related deaths were recorded. One-year outcome is shown in Fig. 3B.

### Performance analysis of TRI-SCORE and comparison with EuroSCORE II and STS-Score

**Fig. 4:**
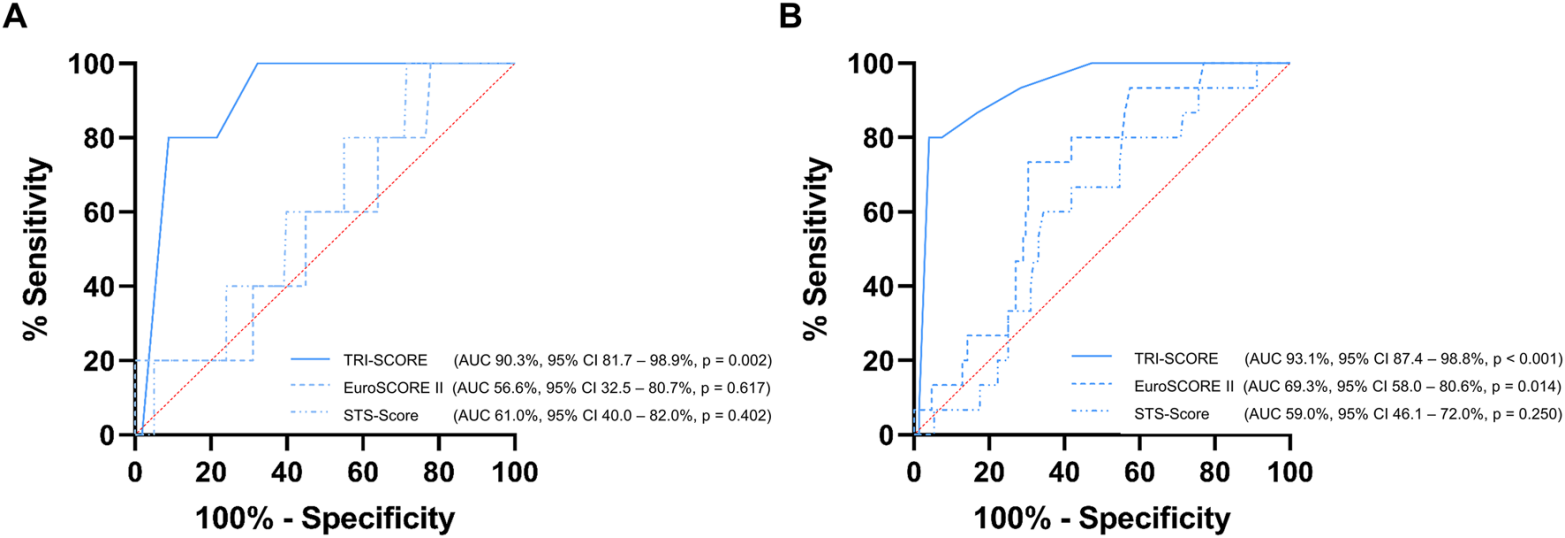
Predictive performance of TRI-SCORE, EuroSCORE II and STS-Score: 30-day mortality (**A**) and one-year mortality (**B**). *AUC* area under the curve, *95% CI* 95% confidence interval

ROC analysis of TRI-SCORE, EuroSCORE II and STS-Score was carried out for 30-day and one-year follow-up periods. TRI-SCORE was a highly reliable tool for the prediction of adverse outcome. AUC for prediction of mortality at 30 days was 90.3% (95% confidence interval (CI) 81.7–98.9%, *p* = 0.002). For one-year mortality, AUC was 93.1% (95% CI 87.4–98.8%, *p* < 0.001). Test performance of TRI-SCORE as assessed by ROC analysis was superior to EuroSCORE II [AUC for 30-day mortality: 56.6% (95% CI 32.5–80.7%, *p* = 0.617), AUC for one-year mortality: 69.3% (95% CI 58.0–80.6%, *p* = 0.014)] and STS-Score [AUC for 30-day mortality: 61.0% (95% CI 40.0–82.0%, *p* = 0.402), AUC for one-year mortality: 59.0% (95% CI 46.1–72.0%, *p* = 0.250)] (Fig. 4). When comparing the effectiveness of risk prediction using net reclassification TRI-SCORE proved superior to both EuroSCORE II (absolute NRI for 30-day and one-year mortality: 24.2%) and STS-Score (absolute NRI for 30-day mortality: 39.4%, absolute NRI for one-year mortality: 41.2%).

## Discussion

T-TEER has emerged as a promising treatment option for patients with symptomatic severe TR and has been shown to reduce mortality and rehospitalization rates in non-randomized trials [9–11]. To date, however, there is no risk prediction tool specifically addressing the complex multimorbid patients undergoing this procedure. With TRI-SCORE the first dedicated mortality risk calculation tool for patients after surgical tricuspid valve procedures has been introduced recently [15]. Our study analyzed the applicability and predictive performance of the original TRI-SCORE for T-TEER patients.

### Risk of adverse outcome increases disproportionately with higher TRI-SCORE

While the original TRI-SCORE has been validated for in-hospital mortality, our study has considered a short-term postprocedural period of 30 days. This reflects the lower ultra-short-term risk of patients undergoing transcatheter procedures compared to surgery. While studies have shown an in-hospital mortality of up to 10% after isolated tricuspid valve surgery [24, 25], no periprocedural death was registered in this present study or in a cohort of 85 patients treated with T-TEER in the TRILUMINATE trial [26].

In our study, TRI-SCORE has shown excellent discriminatory performance, especially in high-risk patients. Strikingly, while differences in risk were only small between low-risk and intermediate-risk patients, mortality increased disproportionately in high-risk patients. In the high-risk group (score ≥ 9), mortality was 21.7% within 30 days and 52.2% throughout the mid-term follow-up. Arguably, the small number of patients in the high-risk group and the low amount with completed mid-term follow-up play a role in risk overestimation. Nonetheless, highly significant differences in outcome were observed already within 30 days after the procedure.

In general, observed mortality rates were lower than predicted in the original model applied to surgical patients only, where a TRI-SCORE of nine or higher predicted an excessive in-hospital mortality of 65% [15]. This might again be due to the markedly higher inherent risk of tricuspid valve surgery.

### TRI-SCORE proves superior to EuroSCORE II and STS-Score

EuroSCORE II and STS-Score are the most broadly used risk assessment tools to predict periprocedural/short-term mortality in patients undergoing cardiac procedures. However, in our analysis their predictive performance after T-TEER was poor and markedly inferior to TRI-SCORE. AUC, measuring the performance of classification models, was consistently higher for the TRI-SCORE when compared to STS-Score or EuroSCORE II, indicating superior discrimination for 30-day and one-year mortality. This relevant difference between the AUCs of the TRI-SCORE and the other scores is supported by not more than marginally overlapping 95% confidence intervals of the AUCs.

To further substantiate the superiority of the TRI-SCORE, net risk reclassification (NRI) was used to compare risk prediction performance of the three scores. Positive absolute NRIs of the TRI-SCORE versus EuroSCORE II (24.2%) or STS-Score (39.4% and 41.2% respectively) demonstrate the TRI-SCORE’s robust superiority in classifying T-TEER patients as low-, intermediate- and high-risk candidates regarding 30-day and one-year mortality. While the advantage of the TRI-SCORE compared to EuroSCORE II has been shown in a larger, multicentric cohort of 313 patients by Omran et al. [16] our data now provide statistic substantiation of superiority over both the EuroSCORE II and the STS-Score.

The inferiority of EuroSCORE II and STS-Score might be explained by their underlying data. The STS-Score has not been validated for tricuspid valve procedures at all [13] and the proportion of tricuspid valve repair surgeries in the development of EuroSCORE II was very low at 4.6% [14]. This implies a severe underrepresentation of tricuspid valve procedures in current score models. Moreover, the complex pathophysiologic reactions of the RV in the context of HF and their prognostic implications are not considered in EuroSCORE II. It is known that RV failure strongly contributes to increased morbidity and mortality in both HF with reduced [27, 28] and preserved ejection fraction [29]. The inclusion of specific parameters of RV failure (clinical signs such as marked peripheral edema, ascites or jugular vein distension, congestive liver disease by elevated bilirubin as well as echocardiographic signs of RV dysfunction) is likely to contribute essentially towards the superior performance of the TRI-SCORE. In our study, TRI-SCORE also showed good precision in the prediction of mid-term mortality—and endpoint that is neglected by both EuroSCORE II and STS-Score.

### Study limitations

There are several limitations to our study that need to be acknowledged. First, our study resembles retrospective, single-center data with a limited number of operating physicians. However, the effectiveness of TR reduction in our cohort was similar to multicenter registries such as the TRILUMINATE trial or the PASTE registry. In TRILUMINATE successful reduction of TR to grade II or lower could be achieved in 60% of patients 30 days after the procedure and in 71% of patients after 1 year [7]. Reported data from the PASTE registry showed 78% of patients with moderate or less TR at discharge and after 1 year [30]. In this present study, 74.5% of patients were discharged with TR grade II or lower. Furthermore, periprocedural risk as assessed by EuroSCORE II was similar in our cohort compared to a large meta-analysis of 771 T-TEER patients with a reported mean score of 6.8 ± 5.4% [31].

Secondly, we report on only a small cohort. Mid-term follow-up was not yet available for a large proportion of patients which is illustrated by a median follow-up of only 168 days. On the other hand, the broadly used clinical risk scores such as EuroSCORE II or STS score are limited to short-term risk prediction only—a timeframe for which we could also show the superiority of the TRI-SCORE. Moreover, given the early and consistent separation of the Kaplan–Meier plots, it is likely that the differences in risk for adverse outcome between the TRI-SCORE quarters will persist over a longer period of time. Nonetheless, a larger multicentric cohort should provide further validation of our data.

## Conclusions

We could show that TRI-SCORE is not only applicable to tricuspid valve surgery but is a valuable tool for short and mid-term risk prediction of mortality following T-TEER. Its performance exceeds the most prominent and established risk scoring systems, the EuroSCORE II and the STS-Score. In the future, TRI-SCORE may be established in clinical decision-making and patient counseling. However, an important question remains: should patients with very high score results be withheld from T-TEER and treated conservatively due to their high predicted short-term mortality, especially since the main cause of death in our cohort was cardiovascular? The therapeutic implications especially of high score results remain to be investigated by larger trials.

## Abbreviations

AUC: Area under the curve
HF: Heart failure
IQR: Interquartile range
NRI: Net reclassification index
RV: Right ventricle
TR: Tricuspid regurgitation
T-TEER: Transcatheter tricuspid edge-to-edge repair
